# The Impact of Different Types of Diet on the Prevention of Diseases among Polish Inhabitants, Including COVID-19 Disease

**DOI:** 10.3390/nu15183947

**Published:** 2023-09-12

**Authors:** Justyna Gołębiowska, Anna Zimny-Zając, Sebastian Makuch, Mateusz Dróżdż, Krzysztof Dudek, Joanna Żórawska, Grzegorz Mazur, Siddarth Agrawal

**Affiliations:** 1 Department and Clinic of Internal Medicine, Occupational Diseases, Hypertension and Clinical Oncology, Wroclaw Medical University, 50-556 Wroclaw, Poland; ju.golebiowska@gmail.com (J.G.); grzegorz.mazur@umw.edu.pl (G.M.); 2 Medonet, Ringier Axel Springer Poland, Domaniewska St. 49, 02-672 Warsaw, Poland; anna.zimny-zajac@medonet.pl; 3 Department of Clinical and Experimental Pathology, Wroclaw Medical University, 50-367 Wroclaw, Poland; sebastian.mk21@gmail.com; 4 Faculty of Medicine, Wroclaw Medical University, J. Mikulicza-Radeckiego 5, 50-345 Wroclaw, Poland; mateuszdrozdz5208@gmail.com; 5 Statistical Analysis Center, Wroclaw Medical University, 50-368 Wroclaw, Poland; krzysztof.dudek@pwr.edu.pl; 6 Clinical Department of Geriatrics, Wroclaw Medical University, Pasteur 4 Street, 50-367 Wroclaw, Poland; joanna.zorawska@umw.edu.pl

**Keywords:** diet, COVID-19, vegetarian, vegan, polish population

## Abstract

Proper nutrition may help in preventing deaths or at least alleviating the symptoms of many chronic diseases. While the COVID-19 disease was still taking its toll, the world had to adjust to new life conditions, which could change nutritional habits. In this observational, cross-sectional study, we aimed to identify the potential correlations between sociodemographic factors and diet and the presence of common chronic diseases among Polish inhabitants. Furthermore, we tried to determine whether the COVID-19 pandemic led to changes in nutritional habits. Therefore, based on the online study (the National Test for Poles’ Health (NTPH), we collected data from 376,102 and 200,000 respondents in two different time frames (before the COVID-19 pandemic: 2019–2020 and during the COVID-19 pandemic: 2021–2022, respectively). Despite the rapid global rise of the COVID-19 pandemic, among our study group, hypertension was still the most commonly occurring disease in both time frames (32.33% in 2019–2020 and 34.95% in 2021–2022, *p* < 0.001). Furthermore, more chronic diseases were reported during the COVID-19 pandemic than in 2019–2020. Regarding sociodemographic factors, male respondents were more likely to develop hypertension and diabetes (OR = 1.35 CI 95% (1.28–1.43), *p* < 0.001; and OR = 1.20 CI 95% (1.11–1.30), *p* < 0.001). Vegetarian diet decreases the likelihood of hypertension, neurological disease, and diabetes (OR = 0.69, CI 95% (0.60–0.81), *p* < 0.001; OR = 0.72, CI 95% (0.59–0.88), *p* = 0.001; and OR = 0.73, CI 95% (0.55–0.96), *p* = 0.026). In line with this, consuming meat meals increases the risk of hypertension (OR = 1.09, CI 95% (1.02–1.17), *p* = 0.009). Interestingly, a reduced-sodium diet has an association with decreased morbidity of COVID-19 disease (OR = 0.72, CI 95% (0.63–0.82), *p* < 0.001). This result brings new light to more research to be done to allow efficient prevention of this disease. In conclusion, our study shows the beneficial role of a balanced diet in reducing the incidence rate of common chronic diseases. Our findings may be educational for those who would like to change their nutritional habits and/or for public health professionals to suggest the implementation of proper diets to their patients.

## 1. Introduction

The question of whether specific types of diets provide health benefits is the subject of many debates and discussions. When considering the right diet, it is worth becoming acquainted with reliable information available in magazines or while surfing the Internet. This is difficult predominantly due to the generation of implausible results by nutrition researchers [1]. Furthermore, due to many sociodemographic factors, including age, budget, access to groceries, and environmental and genetic predispositions, it is almost impossible to generate one potential list of meals that everyone should follow [2]. Therefore, each diet should be adjusted separately for each individual depending on their nutritional needs.

It is worth noting that current literature indicates quite contradictory findings and conclusions regarding each type of diet. Undoubtedly, diet has an impact on the development of disease, but its effects are not always consistent and easy to explain. For instance, it was found that a plant-based diet plays a beneficial role in the prevention of many chronic diseases, including cardiovascular diseases [3] and diabetes [4]. Moreover, it was determined that plant-based meals reduce the risk factors for coronary heart diseases [5,6,7] and body mass index (BMI) [8,9] and lower blood pressure [10,11]. In contrast, Phillips F. suggested that vegetarians and vegans may be at greater risk of increased plasma homocysteine levels, a rising risk factor for cardiovascular disease [12]. In line with this, Ho-Pham et al. suggested that a plant-based diet increases the risk of low bone mineral density, which predisposes individuals to osteoporosis [13]. Furthermore, studies indicate that a vegetarian diet may reduce the risk of certain types of cancer, such as colorectal cancer and breast cancer. For example, a study of 469 Taiwanese women found that a vegetarian diet was inversely associated with breast cancer risk (*p* < 0.05) [14]. However, the evidence regarding the impact of a vegetarian diet on cancer risk is not yet conclusive, and more research is needed in this area. Regarding the role of diet in diabetes, several studies have found that a vegetarian diet can help with the prevention of this disease. For example, a study of over 200,000 adults found that vegetarians had a 53% lower risk of developing diabetes compared to non-vegetarians [15].

The literature is more consistent in the case of a diet containing red meat meals. For instance, Bonaccio et al. proved that consuming this type of food increases the level of crucial biomarkers of inflammation, such as C-reactive protein (CRP), interleukin-6, and fibrinogen [16]. Furthermore, processed meat and red meat that contains many salts (sodium) are positively associated with elevated blood pressure [17] and promote vascular stiffness [18]. In relation to cancer, red meat is rich in carcinogens, including heterocyclic amines and polycyclic aromatic hydrocarbons, which are produced while cooking red meat at high temperatures [19].

Due to the existing COVID-19 pandemic caused by severe acute respiratory syndrome coronavirus 2 (SARS-CoV-2), it is important to take a closer look into different methods of diseases prevention. Undoubtedly, the COVID-19 pandemic has had an impact on almost every corner of an individual’s life. Due to the implemented social isolation and fear of COVID-19 infection, this pandemic at least indirectly impacted changing eating habits. During the highest waves of SARS-CoV-2 infection, many stores and grocery stores were closed. Therefore, due to the limited access to some food products, maintaining a proper diet that is in line with nutritional needs was a big challenge. Furthermore, the economic crisis caused by the COVID-19 pandemic led to an increase in the prices of food products, additionally hindering maintenance of a proper diet. Although COVID-19-associated mortality and morbidity are gradually decreasing [20], some nutritional habits have remained and require improvement. Moreover, there are several pieces of evidence showing the beneficial role of diet in the prevention of COVID-19 disease. For instance, the American Diabetes Association in 2019 and Diabetes Canada in 2020 suggested that low-carbohydrate diets are positively associated with the improvement of glycemia and the reduction of medication doses for people with type 2 diabetes [21,22]. Since it is known that insulin resistance is one of the most important risk factors of COVID-19 severity, it is reasonable to conclude that a low-carbohydrate diet may be useful in preventing COVID-19 disease by increasing glucose levels in the patient’s blood.

Taking into account the abovementioned advantages and disadvantages of different types of diets on health conditions, we put efforts into identifying the correlation between the existing COVID-19 pandemic and the potential changes in nutritional habits among Polish inhabitants. Furthermore, we tried to answer the question of which type of diet may be useful for the prevention of chronic diseases that Polish inhabitants suffer most from. Data included in this study were generated before the COVID-19 pandemic and 2 years after the first outbreak of SARS-CoV-2 infection in December 2019 [23] to show any potential changes in nutritional habits over time.

## 2. Materials and Methods

### 2.1. Study Design

The National Test for Poles’ Health (NTPH)—an online study performed yearly since 2020—is a valuable information source on Polish Internet users’ health that contains data from a large group of Polish Internet users and has been utilized as a foundational tool in the formulation and execution of various studies [24,25]. Thus far, it has been conducted in three waves (2020, 2021, and 2022). For the purpose of this particular study, responses from two waves (2019–2020 and 2021–2022) were analyzed—a representative sample of 376,102 and 200,000 adults, respectively. The goal of this study was to answer the question of whether and which type of diet has a beneficial role in the prevention of COVID-19 disease and other diseases among Polish inhabitants.

The survey was fully anonymous and voluntary [26]. The evaluated sample of the study group was obtained by stratified sampling per the voivodeship demographic structure of Poland. The duration of the survey ranged from 15 to 20 min. All participants provided consent for collecting the data and were informed about the goal of the survey. Participation in the study provided no compensation. The study utilized computer-assisted web interviewing (CAWI) as the primary data collection method. The online questionnaire used in the study regarding diet in the Polish population was translated from Polish to English and is included in Table S1.

### 2.2. Explanatory Variables

The online survey used in the study included questions regarding the respondent’s sociodemographic data (Table S2), past or current diseases (Table S3), and general questions leading to the indication of some nutritional habits (Table S1). These data were self-reported.

Sociodemographic data included: (1) gender (male or female), (2) age, (3) education (primary, secondary, higher), (4) place of residence (village; town, less than 19,000 inhabitants; town, between 20,000 and 49,000 inhabitants; town, between 50,000 and 99,000 inhabitants; town, between 100,000 and 199,000 inhabitants; town, between 200,000 and 499,000 inhabitants; town, more than 500,000 inhabitants), and (5) region of Poland. Furthermore, to determine BMI levels (kg/m^2^), respondents were asked to provide body weight (kg) and body height (cm) (Table 1). The following equation was used to calculate the mean of the BMI value: BMI = body weight (kg)/height (m)^2^ (Table S2).

Based on the answers of the respondents (Table S3), different diseases were taken into consideration, including hypertension, diabetes, heart disease, chronic obstructive pulmonary disease (COPD), allergy and asthma, depression, cancer, joint disease, neurological disease, and COVID-19. These diseases acted as dependent (dichotomous) variables in our study and were self-reported by our study group. These variables were used in logistic regression analysis (presence of the disease (1) or not (0)). Independent variables were related to the type of diet and were limited to participants’ attitudes, behaviors, and preferences regarding food consumed (Table S2). Based on the reliability and honesty of the respondents, 10 diets were analyzed as follows: (1) meals balanced according to the food pyramid, (2) vegetarian meals, (3) vegan meals, (4) meat meals, (5) gluten-free meals, (6) dairy-free meals, (7) carbohydrate-restricted meals, (8) reduced-sodium meals, (9) other types of meals with limited carbohydrates, and (10) other types of sodium-restricted meals. The other analyzed independent variables included in the analysis were (1) the period of the study (before the pandemic/during the pandemic) and sociodemographic factors highlighted above.

### 2.3. Measures

To find out the potential beneficial role of different types of diets in the prevention of chronic diseases and improve health conditions, the survey consisted of several questions regarding the meals eaten most often;, the frequency of eating red meat, fruits, vegetables, junk food, diet supplements, and other over-the-counter medicines; and the frequency of drinking energy drinks. The whole questionnaire is presented in Table S1. The measurement of correlations between sociodemographic factors, diet, and the presence of the COVID-19 pandemic and chronic diseases was carried out based on the participants’ honest and reliable responses, which we believe are consistent with the actual truth. The dichotomization of the answers to questions regarding sociodemographic factors and nutritional habits allowed us to estimate the odds ratios and highlight significant predictors of the presence of common chronic diseases.

### 2.4. Statistics

Nominal and ordinal variables are presented in the contingency tables as numbers (n) and percentages (%). Adjusted beta coefficients (β), odds ratios (OR), and 95% confidence intervals (95% CI) are reported for regression analysis; *p* < 0.05 is considered statistically significant. Software package STATISTICA v. 13.3 (TIBCO Software Inc., Palo Alto, CA, USA) was used for the analysis.

## 3. Results

### 3.1. Study Group

To observe the potential association of different nutritional habits, sociodemographic factors, and the presence of the COVID-19 pandemic with the likelihood of developing chronic diseases among Polish inhabitants, we analyzed data collected at the beginning of the pandemic (2019–2020) and during (2021–2022) the pandemic. In 2019–2020, no patient with COVID-19 was detected; the outbreak of the pandemic occurred in December 2019. Therefore, comparing data from 2019–2020 and 2021–2022 may show significant differences regarding nutrition habits in these two COVID-19-pandemic time frames and their consequences for health conditions.

In 2019–2020, a total of 376,102 participants were included in the study—58.3% women and 41.7% men. The mean age was 48 years old (SD = 15 years). The majority of the respondents had higher education (39.3%). The place of residence was quite evenly distributed; a similar percentage of the study group lived either in the village or in a large city with more than 500,000 inhabitants (21.3% and 21.6%, respectively). Respondents were mostly living in the Mazovian and Silesian voivodeships (16.5% and 14.2%, respectively). The mean BMI was 26.9 kg/m^2^. According to the Centers of Disease Control and Prevention (CDC) guidelines, these participants fall into the lower limit of overweight [27]. Further characteristics of the subjects included in this study can be found in Table S2.

In contrast, in 2021–2022, a total of 200,000 Polish inhabitants participated in the study—59.6% women and 40.4% men. The mean age in these years was 53 years old with SD = 15 years. The majority of the participants had higher education (36.6%). The place of residence was quite evenly distributed; nevertheless, a higher percentage of respondents were living in villages (22.1%). Similar to previous years, the Mazovian and Silesian voivodeships were the ones most occupied by our study population (15.8% and 14.1%, respectively). The mean BMI was 27.2. According to CDC guidelines, these participants fall into the lower limit of overweight [27]. Further characteristics of the subjects included in this study can be found in Table S2.

Due to the very large number of respondents in the compared periods (376,102 vs. 200,000), the statistical analysis was carried out on representative random samples taken from both populations. Assuming a significance level of α = 0.01 and a permissible error of e = 1%, the minimum sample size for the period before the pandemic (2019–2020) was 15,887, whereas for the period during the pandemic (2021–2022) it was 15,317. The same number of respondents was assumed to be 17,000 in both periods. In both samples, the appropriate structure in terms of gender and age was obtained using proportional stratified sampling. The results of the comparisons are presented in Table 1. Both samples were homogeneous in terms of all sociodemographic characteristics (*p* > 0.05).

### 3.2. Observed Diseases

**Figure 1:**
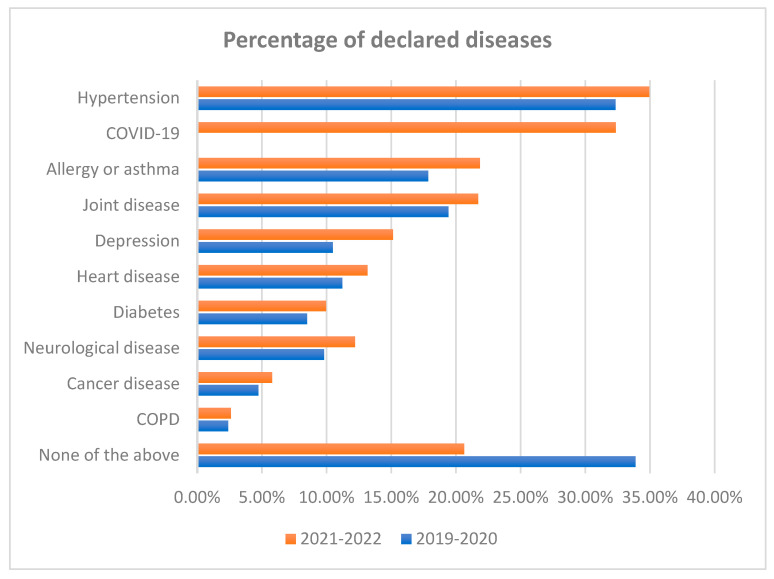
Percentage of self-reported diseases before the COVID-19 pandemic (2019–2020) and during the pandemic (2021–2022).

In both the years 2019–2020 and 2021–2022, hypertension was the most commonly occurring disease in our study population (32.33% and 34.95%, respectively, *p* < 0.001). In 2021–2022, the second position belonged to COVID-19 disease (32.5%, *p* < 0.001), whereas in 2019–2020, it belonged to joint disease (19.41%, *p* < 0.001). In 2021–2022, the third most present disease was allergies and asthma (21.85%, *p* < 0.001). It is worth noting there is a relatively high percentage of different diseases that are not analyzed in this study (none of the above: 33.89% in 2019–2020 and 20.64% in 2021–2022, respectively). Furthermore, each disease occurred statistically more often in 2021–2022 than in 2019–2020, which was mostly visible in respondents suffering from depression, allergies, and asthma (Figure 1, Table 2). This analysis was carried out on representative random samples taken from both populations (before the COVID-19 pandemic: 2019–2020 and during the COVID-19 pandemic: 2021–2022). The data from all respondents involved in this study are shown in Table S3.

### 3.3. Types of Diet

In both time periods (2019–2020 and 2021–2022), most of the respondents declared that they consumed meat meals and meals balanced according to the food pyramid (13.51% and 11.14%, *p* < 0.001; and 12.16% and 10.64%, *p* < 0.001, respectively, Table 3). The statistical difference between two analyzed time periods was also observed among carbohydrate-restricted meals (2.29% and 2.56%, *p* = 0.023, respectively), reduced-sodium meals (3.75% and 3.21%, *p* < 0.001, respectively), and other types of meals (0.89% and 1.11%, *p* = 0.005, respectively). In 2021–2022, more respondents answered that they did not know which type of diet they ate the most, assuming that they did not pay that much attention to their food consumption (18.07%, *p* < 0.001). It is worth mentioning that this analysis was carried out on representative random samples taken from both populations (before the COVID-19 pandemic and during the COVID-19 pandemic).

From a total of 200,000 respondents, 82,233 of them declared that they ate red meat once or twice a month (41.2%, Table S4). A slightly lower number of respondents answered that they ate red meat one to three times a week (36.8%, Table S4). Furthermore, more than half of the respondents reported that they had never been on a slimming diet (112,563/200,000; 56.3%, Table S4). A total of 43.2% of respondents reported eating junk food such as hamburgers, fries, hot dogs, etc. less than once a month (86,395/200,000, Table S4), whereas 245 people ate this kind of food every day (0.12%, Table S4). A total of 27.7% and 78.5% of respondents avoid drinking sweetened, carbonated or non-carbonated beverages and energy drinks, respectively (55,442/200,000 and 156,954/200,000, Table S4). Our study population ate more vegetables than fruits several times a week (84,200/200,000; 42.1% and 72,400/200,000; 36.2%, respectively) but more fruits than vegetables every day (84,400/200,000, 42.2% and 70,600/200,000, 35.3%). A total of 40.1% of respondents had not taken any dietary supplements or other over-the-counter drugs in the previous year (in 2020). The other group (59.9%) was asked for the reason behind taking these medicines. The most frequent answers were to enrich their body with vitamins (35.2%) and to improve the condition of their hair and/or skin (14.0%) (Table S4).

In Table 4, we determined the percentage of respondents in groups differing in terms of the type of consumed diet and self-reported disease in 2021–2022. For instance, among 38.2% of respondents with hypertension (Table S3), 13.75% of them were consuming another type of sodium-restricted meals. This type of diet was also consumed in 11.41% of respondents with COVID-19 disease (Table 4).

To identify the potential correlation of diet, sociodemographic factors, and the presence of the COVID-19 pandemic with the likelihood of suffering from common chronic diseases, we focused on the analysis of data of 200,000 respondents who filled in the questionnaire in 2021–2022 (Table S3). In these particular years, the COVID-19 pandemic was still taking its toll, but it was an integral part of society that everyone had to deal with. Therefore, we think that this is the best year to describe the more stable changes in the diet caused by the COVID-19 pandemic rather than the unexpected outbreak of this disease.

### 3.4. Correlation between the Presence of COVID-19 Pandemic, Sociodemographic Factors, and Diet and Hypertension

During the COVID-19 pandemic (2021–2022), hypertension was observed in 76,441 (38.2%) of all respondents (76,441/200,000, 38.2%, *p* < 0.001, Table S3). To estimate the probability of developing hypertension, the following mathematical model in the logit form was used:

Logit P{Hypertension = 1|X} = −7.57 + 0.13 × during the pandemic + 0.30 × male sex + 0.07 × age − 0.08 × education level − 0.02 × population of the place of residence + 0.12 × BMI − 0.14 × balanced meals − 0.37 × vegetarian meals + 0.09 × meat meals + 0.33 × reduced-sodium meals

Factors that increased the likelihood of hypertension were the COVID-19 pandemic period (OR = 1.14, CI 95% (1.08–1.20), *p* < 0.001), male gender (OR = 1.35 CI 95% (1.28–1.43), *p* < 0.001), older age (OR = 1.07 CI 95% (1.07–1.07), *p* < 0.001), high BMI (OR = 1.13, CI 95% (1.12–1.14), *p* < 0.001), meat meals (OR = 1.09, CI 95% (1.02–1.17), *p* = 0.009), and reduced-sodium meals (OR = 1.14, CI 95% (1.08–1.20), *p* < 0.001). In contrast, highly educated (OR = 0.93, CI 95% (0.89–0.96), *p* < 0.001) respondents living in cities with a large population (OR = 0.99, CI 95% 0.97–1.00), *p* = 0.014) were less likely to suffer from hypertension, as well as those consuming meals balanced according to the food pyramid (OR = 0.87, CI 95% (0.81–0.93), *p* < 0.001) and vegetarian meals (OR = 0.69, CI 95% (0.60–0.81), *p* < 0.001). Other analyzed factors were not included in the model due to not-significant differences. The values of the regression coefficients for the correlation of diet, sociodemographic factors, and the presence of the COVID-19 pandemic with hypertension can be observed in Table 5.

### 3.5. Correlation between Sociodemographic Factors, Diet, and COVID-19 Disease

In 2021–2022, COVID-19 disease was observed in 62,414 of all analyzed respondents (62,414/200,000, 31.2%, *p* < 0.001, Table S3). To estimate the probability of developing COVID-19 disease, the following mathematical model in the logit form was used:

logit P{COVID-19 = 1|X} = 1.461 − 0.122 × male gender − 0.011 × age + 0.193 × marital status − 0.199 × meals balanced according to the food pyramid − 0.33 × reduced-sodium meals

Regarding the sociodemographic factors, married respondents were more likely to develop COVID-19 infection (OR = 1.24, CI 95% (1.14–1.29), *p* < 0.001). There was no association between diet and the increased likelihood of suffering from COVID-19 disease among our study group. However, it was determined that male and young respondents were less likely to exhibit COVID-19 symptoms (OR = 0.88, CI 95% (0.83–0.94) *p* < 0.001; and OR = 0.99, CI 95% (0.99–0.99) *p* < 0.001, respectively). However, maintaining proper nutritional habits while consuming meals according to the food pyramid decreased the likelihood of suffering from COVID-19 infection (OR = 0.82, CI 95% (0.76–0.89), *p* < 0.001). Furthermore, we found that respondents declaring the consumption of reduced-sodium meals were also less likely to develop COVID-19 infection (OR = 0.72, CI 95% (0.63–0.82), *p* < 0.001). Other analyzed factors were not included in the model due to not-significant differences. The values of the regression coefficients for the correlation of diet and sociodemographic factors with COVID-19 infection can be observed in Table 6.

### 3.6. Correlation between the Presence of the COVID-19 Pandemic, Sociodemographic Factors, and Diet, Allergies, and Asthma

In 2021–2022, allergies and asthma were observed in 43,000 of all analyzed respondents (43,000/200,000, 21.5%, *p* < 0.001, Table S3). To estimate the probability of developing allergies and asthma, the following mathematical model in the logit form was used:

logit P{allergies and asthma = 1|X} = −1.440 + 0.258 × during the pandemic − 0.362 × male gender − 0.013 × age − 0.153 × marital status + 0.036 × population of the place of residence + 0.014 × BMI + 0.198 × vegetarian meals − 0.951 × gluten-free meals − 0.412 × dairy-free meals

Factors that increased the likelihood of allergies and asthma were as follows: (1) the COVID-19 pandemic period (OR = 1.29, CI 95% (1.23–1.37), *p* < 0.001), (2) living in big cities (OR = 1.04, CI 95% (1.02–1.05), *p* < 0.001), and (3) high BMI (OR = 1.01, CI 95% (1.01–1.02), *p* < 0.001). Furthermore, we found an association between the increased probability of suffering from allergies and asthma among respondents who declared that they consumed vegetarian meals and dairy–free meals (OR = 1.22, CI 95% (1.08–1.37), *p* = 0.001; and OR = 1.51, CI 95% (1.13–2.01), *p* = 0.005, respectively). In contrast, there was less likelihood of suffering from allergies and asthma among male, young, and married respondents (OR = 0.7, CI 95% (0.66–0.74), *p* < 0.001; OR = 0.99, CI 95% (0.98–0.99), *p* < 0.001; and OR = 0.86, CI 95% (0.81–0.91), *p* < 0.001, respectively). Furthermore, we did not determine the specific type of diet that decreased the likelihood of suffering from allergies and asthma. Other analyzed factors were not included in the model due to not-significant differences. The values of the regression coefficients for the correlation of diet, sociodemographic factors, and the presence of the COVID-19 pandemic with allergies and asthma can be observed in Table 7.

### 3.7. Correlation between the Presence of the COVID-19 Pandemic, Sociodemographic Factors, and Diet and Joint Diseases

During the COVID-19 pandemic (2021–2022), joint disease was observed in 48,086 of all respondents (48,086/200,000, 24.0%, *p* < 0.001, Table S3). To estimate the probability of suffering from joint disease, the following mathematical model in the logit form was used:

logit P{joint disease = 1|X} = −4.351 + 0.117 × during the pandemic − 0.548 × male gender − 0.053 × age − 0.342 × education level −0.06 × marital status − 0.017 × population of the place of residence + 0.045 × BMI − 0.102 × meals balanced according to the food pyramid + 0.43 × dairy-free meals

Factors that increased the likelihood of joint disease were as follows: (1) the COVID-19 pandemic period (OR = 1.12, CI 95% (1.06–1.19), *p* < 0.001), (2) older age (OR = 1.05, CI 95% (1.05–1.06), *p* < 0.001), and (3) high BMI (OR = 1.05, CI 95% (1.04–1.05), *p* < 0.001). Furthermore, we found an association with increased probability of suffering from joint disease among respondents who declared that they consumed dairy-free meals (OR = 1.41, CI 95% (1.03–1.91), *p* = 0.001). In contrast, there was less likelihood of suffering from joint disease among male respondents, those with high education levels, those who were married, and those who lived in big cities (OR = 0.58, CI 95% (0.54–0.61), *p* < 0.001; OR = 0.71, CI 95% (0.68–0.74), *p* < 0.001; OR = 0.94, CI 95% (0.89–1.00), *p* < 0.001), and OR = 0.98, CI 95% (0.97–1.00), respectively). Moreover, according to our study group, respondents who consumed meals balanced according to the food pyramid were less likely to have joint disease symptoms (OR = 0.9, CI 95% (0.84–0.97), *p* = 0.006). Other analyzed factors were not included in the model due to not-significant differences. The values of the regression coefficients for the correlation of diet, sociodemographic factors, and the presence of the COVID-19 pandemic with joint disease can be observed in Table 8.

### 3.8. Correlation between the Presence of the COVID-19 Pandemic, Sociodemographic Factors, and Diet and Depression

During the COVID-19 pandemic (2021–2022), depression was observed in 30,277 of all respondents (30,277/200,000, 15.1%, *p* < 0.001, Table S3). To estimate the probability of suffering from depression, the following mathematical model in the logit form was used:

logit P{depression = 1|X} = −1.326 + 0.424 × during the pandemic − 0.556 × male gender − 0.005 × age − 0.141 × education level − 0.396 × marital status + 0.053 × population of the place of residence − 0.185 × meals balanced according to the food pyramid + 0.25 × vegetarian food

Factors increasing the likelihood of depression were as follows: (1) the COVID-19 pandemic period (OR = 1.53, CI 95% (1.43–1.63), *p* < 0.001), (2) living in a big city (OR = 1.05, CI 95% (1.04–1.07), *p* < 0.001), and (3) the consumption of vegetarian meals (OR = 1.28, CI 95% (1.12–1.47), *p* < 0.001). In contrast, regarding sociodemographic factors, male gender, older age, education level, and married status decreased the likelihood of depression (OR = 0.57, CI 95% (0.53–0.62), *p* < 0.001; OR = 0.99, CI 95% (0.99–1.00), *p* < 0.001; OR = 0.87, CI 95% (0.83–0.91); and OR = 0.67, CI 95% (0.63–0.72), *p* < 0.001, respectively). Furthermore, respondents who declared that they consumed meals balanced according to the food pyramid were less likely to suffer from depression (OR = 0.83, CI 95% (0.76–0.91), *p* < 0.001). Other analyzed factors were not included in the model due to not-significant differences. The values of the regression coefficients for the correlation of diet, sociodemographic factors, and the presence of the COVID-19 pandemic with depression can be observed in Table 9.

### 3.9. Correlation between the Presence of the COVID-19 Pandemic, Sociodemographic Factors, and Diet and Heart Disease

During the COVID-19 pandemic (2021–2022), heart disease was observed in 30,400 of all respondents (30,400/200,000, 15.2%, *p* < 0.001, Figure 1, Table 2).To estimate the probability of suffering from heart disease, the following mathematical model in the logit form was used:

logit P{heart disease = 1|X} = −5.424 + 0.164 × during the pandemic + 0.058 × age − 0.18 × education level + 0.023 × BMI − 0.116 × meals balanced according to the food pyramid + 0.505 × gluten-free meals + 0.529 × dairy-free meals + 0.182 × reduced-sodium meals

Factors increasing the likelihood of heart disease were as follows: (1) the COVID-19 pandemic period (OR = 1.18, CI 95% (1.10–1.26), *p* < 0.001), (2) older age (OR = 1.06, CI 95% (1.06–1.06), *p* < 0.001), and (3) high BMI (OR = 1.02, CI 95% (1.02–1.03). Regarding nutritional habits, we found that gluten-free meals, dairy-free meals, and reduced-sodium meals increased the likelihood of heart disease (OR = 1.66, CI 95% (1.21–2.28), *p* = 0.002; OR = 1.7, CI 95% (1.19–2.43), *p* = 0.004; and OR = 1.2 CI 95% (1.06–1.35), *p* = 0.003, respectively). In contrast, high education level and the consumption of meals balanced according to the food pyramid decreased the likelihood of developing heart disease (OR = 0.84, CI 95% (0.80–0.88), *p* < 0.001; and OR = 0.89, CI 95% (0.81–0.98), *p* = 0.016). Other analyzed factors were not included in the model due to not-significant differences. The values of the regression coefficients for the correlation of diet, sociodemographic factors, and the presence of COVID-19 pandemic with heart disease can be observed in Table 10.

### 3.10. Correlations between Neurological Disease and Diet

During the COVID-19 pandemic (2021–2022), neurological disease was observed in 26,075 respondents (26,075/200,000; 13.0%, *p* < 0.001, Table S3). To estimate the probability of suffering from neurological disease, the following mathematical model in the logit form was used:

logit P{neurological disease = 1|X} = −2.548 + 0.220 × during the pandemic − 0.377 × male gender + 0.026 × age − 0.365 × education level − 0.126 × marital status − 0.018 × population of the place of residence − 0.207 × meals balanced according to the food pyramid − 0.329 × vegetarian meals + 0.456 × dairy-free meals

Factors increasing the likelihood of neurological disease were as follows: (1) the COVID–19 pandemic period (OR = 1.25, CI 95% (1.16–1.34), *p* < 0.001), (2) older age (OR = 1.03, CI 95% (1.02–1.03), *p* < 0.001), and (3) dairy–free meals (OR = 1.58, CI 95% (1.12–2.22), *p* < 0.001). On the other hand, factors that reduced the probability of developing neurological diseases were (1) male gender (OR = 0.69, CI 95% (0.64–0.74), *p* < 0.001), (2) high education level (OR = 0.69, CI 95% (0.66–0.73, *p* < 0.001), (3) married marital status (OR = 0.88, CI 95% (0.82–0.95), *p* = 0.001), and (4) living in big cities (OR = 0.98, CI 95% (0.97–1.00), *p* < 0.027). Furthermore, regarding the type of diet, among our study group we proved that those who consumed meals balanced according to the food pyramid and vegetarian meals were less likely to develop neurological diseases (OR = 0.81, CI 95% (0.74–0.89), *p* < 0.001; and OR = 0.72, CI 95% (0.59–0.88), *p* = 0.001, respectively). Other analyzed factors were not included in the model due to not-significant differences. The values of the regression coefficients for the correlation of diet, sociodemographic factors, and the presence of the COVID-19 pandemic with neurological disease can be observed in Table 11.

### 3.11. Correlation between the Presence of the COVID-19 Pandemic, Sociodemographic Factors, and Diet and Diabetes

During the COVID-19 pandemic (2021–2022), diabetes was observed in 22,784 respondents (22,784/200,000, 11.4%, *p* < 0.001, Table S3). To estimate the probability of developing diabetes, the following mathematical model in the logit form was used:

logit P{diabetes = 1|X} = −8.339 + 0.146 × during the pandemic + 0.184 × male gender + 0.054 × age − 0.19 × education level + 0.091 × marital status + 0.02 × population of the place of residence + 0.114 × BMI − 0.315 × vegetarian food − 1.078 × dairy-free meals

Factors that increased the likelihood of diabetes were (1) the COVID-19 pandemic period (OR = 1.16, CI 95% (1.07–1.25), *p* < 0.001), (2) male gender (OR = 1.20 CI 95% (1.11–1.30), *p* < 0.001), (3) older age (OR = 1.06 CI 95%(1.05–1.06), *p* < 0.001), and (4) high BMI (OR = 1.12, CI 95% (1.11–1.13), *p* < 0.001). Regarding other sociodemographic variables, married respondents living in big cities were more likely to suffer from diabetes (OR = 1.10, CI 95% (1.01–1.19), *p* = 0.028; and OR = 1.02, CI 95% (1.00–1.04), *p* = 0.031, respectively). In contrast, highly educated respondents and those declaring that they consumed a vegetarian diet or dairy-free meals were less likely to develop diabetes (OR = 0.83 CI 95% (0.78–0.87), *p* < 0.001; OR = 0.73, CI 95% (0.55–0.96), *p* = 0.026; and OR = 0.34, CI 95% (0.16–0.74), *p* = 0.007, respectively). Other analyzed factors were not included in the model due to not-significant differences. The values of the regression coefficients for the correlation of diet, sociodemographic factors, and the presence of the COVID-19 pandemic with diabetes can be observed in Table 12.

### 3.12. Correlation between the Presence of the COVID-19 Pandemic, Sociodemographic Factors, and Diet and Cancer

During the COVID-19 pandemic (2021–2022), cancer was observed in 14,000 respondents (14,000/200,000, 7%, *p* < 0.001, Table S3). To estimate the probability of developing cancer, the following mathematical model in the logit form was used:

logit P{cancer = 1|X} = −6.025 + 0.199 × during the pandemic − 0.456 × male gender + 0.053 × age + 0.035 × population of the place of residence

Among our study group, factors that increased the likelihood of cancer were (1) the COVID-19 pandemic period (OR = 1.22, CI 95% (1.11–1.34), *p* < 0.001), (2) older age (OR = 1.05 CI 95% (1.05–1.06), *p* < 0.001), and (3) living in a big city (OR = 1.04, CI 95% (1.01–1.06), *p* = 0.002). In contrast, male respondents were less likely to develop cancer (OR = 0.63, CI 95% (0.57–0.7), *p* < 0.001). We did not find any association between the development of cancer and diet. The values of regression coefficients for the correlation of sociodemographic factors and the presence of the COVID-19 pandemic with cancer can be observed in Table 13.

### 3.13. Correlation between the Presence of the COVID-19 Pandemic, Sociodemographic Factors, and Diet and COPD

During the COVID-19 pandemic (2021–2022), COPD was observed in 6812 of the respondents (6812/200,000, 3.4%, *p* < 0.001, Table S3). To estimate the probability of developing COPD, the following mathematical model in the logit form was used:

logit P{COPD = 1|X} = −6.856 + 0.071 × age + 0.015 × BMI − 0.495 × education level − 0.658 × meals balanced according to the food pyramid

Factors that increased the likelihood of COPD were older age and high BMI (OR = 1.07, CI 95% (1.07–1.08), *p* < 0.001; and OR = 1.02, CI 95% (1.00–1.03), *p* = 0.037, respectively). In contrast, high education and the consumption of meals balanced according to the food pyramid decreased the likelihood of developing COPD (OR = 0.61, CI 95% (0.55–0.67), *p* < 0.001; and OR = 0.52, CI 95% (0.41–0.65), *p* < 0.001, respectively). Other analyzed factors were not included in the model due to not-significant differences. The values of the regression coefficients for the correlation of diet, sociodemographic factors, and the presence of the COVID-19 pandemic with COPD can be observed in Table 14.

## 4. Discussion

According to the World Health Organization, noncommunicable diseases such as heart disease, stroke, cancer, diabetes, and chronic lung disease are the reason behind approximately 41 million deaths per year, which account for 71% of all deaths worldwide [28]. This statistical percentage in Poland is not lower at all. As shown by the findings of the National Health Test of Poles 2022, approximately every third Polish inhabitant is diagnosed with hypertension, every fifth person suffers from allergies or asthma, and every sixth person develops depression [29]. Consistently, our study shows high incidence rates of hypertension (32.33% in 2019–2020 and 34.95% in 2021–2022), COVID-19 infection (32.35% in 2021–2022), and joint disease (19.41% in 2019–2020 and 21.71% in 2021–2022). Unfortunately, these incidence rate percentages were higher in 2021–2022 than in 2019–2020, which is in line with the National Health Test Poles 2022 [29]. Furthermore, we observed a significant correlation between the presence of the COVID-19 pandemic and increased likelihood of developing one of the analyzed chronic diseases.

Our study revealed that male respondents were more likely to develop hypertension (OR = 1.35 CI 95% (1.28–1.43), *p* < 0.001, Table 5). This result is consistent with several other studies [30,31,32,33]. However, it is worth taking into account the age of the analyzed respondents. Di Giosia et al. reported that postmenopausal women are more likely to display a rapid increase in hypertension prevalence. Several studies suggest that sex hormone changes play a crucial role in the pathophysiology of hypertension in women after menopause [30,31]. For instance, data from the US show a higher incidence of hypertension among male respondents until the age of 45 years. After this age, a similar rate was observed, and hypertension was more prevalent in women than in men after the age of 65 years [31]. Furthermore, our study revealed that male respondents are more likely to suffer from diabetes (OR = 1.20 CI 95% (1.11–1.30), *p* < 0.001, Table 12). Although this finding is quite contradictory to the known literature [34], it is worth considering other factors that increase the susceptibility to morbidity to chronic diseases, including BMI, stress level, diet, physical activity, past or current secondary diseases, etc. Nevertheless, our study shows that sex affects the probability of morbidity to different chronic diseases. However, additional studies examining the mechanisms of pathophysiology of sex differences of several chronic diseases are required.

There was a significant association between high education levels and reduced likelihood of suffering from almost all of the analyzed diseases (we did not see this correlation only with COPD, allergies and asthma, and COVID-19 disease). This finding shows the significant role of awareness in the prevention of common diseases. Furthermore, as documented by Cutler and lleras-Muney, individuals with higher education levels obtained more flu shots, vaccines, mammograms, Pap smears, and colonoscopies [35]; the earlier a disease is detected, the greater the chance of survival. Therefore, clinical healthcare professionals and several laws should initiate health promotion strategies to increase the attention and awareness among people [36].

The specific impact of different diets on preventing SARS-CoV-2 infection and the overall management of COVID-19 disease remains a debatable and scarce topic of research. This study aimed to investigate the potential beneficial roles of various diet types in the prevention of common 21st-century diseases, including COVID-19, as well as to explore the association between the existence of the COVID-19 pandemic and potential changes in nutritional habits among the Polish population. We found a significant association between the consumption of meals according to the food pyramid and decreased likelihood of developing hypertension (Table 5), COVID-19 disease (Table 6), joint disease (Table 8), depression (Table 9), heart disease (Table 10), and neurological diseases (Table 11). This finding means that diet plays a role in preventing these diseases [37].

A study conducted by Franco et al. analyzed the impact of COVID-19 confinement on physical activity and Mediterranean diet adherence among employees participating in a Healthy Cities program in Spain [38]. The results showed increased sedentary behavior but also higher levels of physical activity and greater adherence to the Mediterranean diet during the pandemic, suggesting a positive effect of remote work on health-promotion efforts among this population.

In another systematic review that examined changes in eating behavior during the COVID-19 pandemic by comparing behaviors before and after its outbreak, the findings revealed shifts towards increased snack frequency, a preference for sweets and ultra-processed foods over fruits and vegetables, and higher alcohol consumption across different countries [39]. Consequently, adherence to healthy diets decreased, emphasizing the importance of considering these findings for future policies and strategies during similar alarming situations like the COVID-19 pandemic.

Proper nutrition has long been recognized as a key factor in preventing various diseases and alleviating their symptoms. There is mounting evidence that suggests that a vegetarian diet can have a significant impact on disease prevention. Studies indicate that a vegetarian diet may reduce the risk of certain types of cancer, such as colorectal cancer and breast cancer. Another study found that a vegetarian diet was associated with better glycemic control in individuals with type 2 diabetes [15]. In addition to the above, a vegetarian diet has also been associated with a lower risk of obesity, hypertension, and chronic kidney disease [40,41,42]. It is thought that the high fiber and low fat content of a vegetarian diet may contribute to these health benefits. A systematic review and meta-analysis of nine studies found that individuals who followed a plant-based diet had a lower risk of developing type 2 diabetes compared to individuals who did not follow this kind of diet [43]. A more recent systematic review and meta-analysis of 11 studies found that individuals with type 2 diabetes who followed a vegetarian diet for at least 12 weeks had decreased body weight and improved glycemia [44]. These findings suggest that a vegetarian diet may be effective in preventing type 2 diabetes. Consistently, our study revealed the significant association between the consumption of a plant-based diet and decreased likelihood of developing hypertension (Table 5), diabetes (Table 12), and neurological diseases (Table 11). In line with these results, hypertension was less common among respondents who declared that they consumed meat meals (Table 5). Furthermore, our study found that respondents with a plant-based diet were more likely to have depression symptoms (Table 9). This finding is in line with the study by Jain et al. who reviewed data from 23 studies, including 25 study outcomes. Most of the studies (44%) proved a significant association with higher rates of depression; seven outcomes (28%) showed a beneficial role of this diet on depression. The other seven outcomes revealed no correlation in this regard. These pieces of evidence demonstrate contradictory results, possibly due to the heterogeneity of the studies analyzed [45].

Interestingly, our study shows a significant correlation between a decreased risk of COVID-19 infection and the consumption of reduced-sodium meals (Table 6). To the best of our knowledge, there is no study describing this association between this particular type of diet and the prevalence of COVID-19 infection. Therefore, this sheds new light in the scientific world that we believe is worth investigating in detail. Sodium plays a crucial role in the regulation of electrolytic balance and the expression of ACE in SARS-CoV-2. As Luo et al. and Lippi et al. found, sodium concentration is lowered in COVID-19 patients and decreases in line with the severity of the disease [46,47]. In contrast, Cure et al. proved that COVID-19 infection may increase the risk of hyperglycemia through the glucose regulation by the Na+/H+ exchanger and lactate pathways. During COVID-19 infection, the activated angiotensin II contributes to insulin resistance and leads to hypoxia and extracellular acidification. The consequence of this mechanism is the accumulation of calcium and sodium ions in the cells and the production of reactive oxygen species, which damage pancreatic tissues [48].

The present study is subject to several notable limitations. Initially, the reliance on an online survey as the primary data collection method introduces potential concerns regarding the authenticity of participant responses. The absence of direct oversight makes it challenging to ascertain the accuracy and sincerity of the information provided by respondents. These concerns refer to self-reported sociodemographic data, diagnosis of chronic diseases, and diet. Moreover, the extent of incompleteness or dropout rates at different stages of the research remains uncertain, thus potentially impacting the overall quality and reliability of the collected data. Additionally, the investigation’s focus on food preferences lacks a critical analysis of the caloric content of the diets consumed by participants, rendering the impact of dietary choices on the outcomes less comprehensive. This omission is particularly significant, as differences in metabolism between genders could yield disparate physiological responses to diet, thus introducing a potential confounding variable that may have affected the study’s conclusions.

## 5. Conclusions

This observational, cross-sectional study highlights the potential beneficial roles of a balanced diet according to the food pyramid in preventing common diseases, such as hypertension, COVID-19, and joint, heart, and neurological diseases. Moreover, the COVID-19 pandemic increased the likelihood of developing other diseases. Nevertheless, the direct association between these variables has to be analyzed in further studies. Furthermore, our study revealed that a plant-based diet decreases the likelihood of suffering from hypertension, neurological diseases, and diabetes. Interestingly, a sodium-restricted diet is likely to decrease the incidence rates of COVID-19 infection, an association that has to be tested in detail. These findings provide valuable educational insights for individuals seeking to make informed decisions about their dietary habits, as well as for public health professionals wanting to implement several strategies in nutritional habits based on the health conditions of their patients.

## Figures and Tables

**Table 1:** Sociodemographic characteristics of the respondents (*p* < 0.05 was considered statistically significant).

Features (Variables)	Before the Pandemic 2019–2020	During the Pandemic 2021–2022	*p*-Value
Sex:			0.974
Male	40.63%	40.61%	
Female	59.37%	59.39%	
Age (years)			0.170
M ± SD	50 ± 14	50 ± 14	
Me [Q1; Q3]	50 [40; 61]	50 [40; 61]	
Min–Max	18–99	18–93	
Level of education:			0.066
Primary education	11.54%	12.35%	
Secondary education	39.39%	39.20%	
Higher education	49.06%	48.45%	
Status			0.058
Single	14.84%	15.65%	
In a relationship (not married)	18.92%	18.10%	
Married	61.09%	60.89%	
Widow/widower	5.15%	5.36%	
Place of residence:			0.062
Village	20.95%	20.89%	
Town, less than 19,000 inhabitants	11.36%	11.82%	
Town, between 20,000 and 49,000 inhabitants	14.27%	14.89%	
Town, between 50,000 and 99,000 inhabitants	11.24%	11.34%	
Town, between 100,000 and 199,000 inhabitants	10.31%	10.71%	
Town, between 200,000 and 499,000 inhabitants	10.53%	10.28%	
Town, more than 500,000 inhabitants	21.33%	20.08%	
Country region			
Central region	9.49%	9.09%	0.204
Southern region	24.11%	23.47%	0.166
Eastern region	11.84%	11.59%	0.474
Northwestern region	15.20%	15.83%	0.109
Southwestern region	10.18%	10.55%	0.263
Northern region	13.38%	13.88%	0.179
Mazovian district	15.80%	15.59%	0.594
Body height (cm)			0.660
M ± SD	170 ± 9	170 ± 9	
Me [Q1; Q3]	170 [164; 176]	170 [164; 176]	
Min–Max	130–206	140–205	
Body mass (kg):			0.468
M ± SD	79 ± 17	79 ± 18	
Me [Q1; Q3]	78 [65; 90]	77 [65; 90]	
Min–Max	30–190	33–205	
BMI (kg/m^2^):			0.065
M ± SD	27.1 ± 5.0	27.1 ± 5.2	
Me [Q1; Q3]	27 [24; 30]	26 [24; 30]	
Min–Max	13–60	13–59	

**Table 2:** Percentage of observed diseases (*p* < 0.05 is considered statistically significant).

Observed Diseases	2019–2020 * n = 17,000	2021–2022 *n* = 17,000	*p*-Value
1. Hypertension	32.33%	34.95%	<0.001
2. Diabetes	8.49%	9.95%	<0.001
3. Heart disease	11.22%	13.16%	<0.001
4. Chronic obstructive pulmonary disease (COPD)	2.39%	2.61%	0.198
5. Allergies or asthma	17.86%	21.85%	<0.001
6. Depression	10.48%	15.14%	<0.001
7. Cancer disease	4.74%	5.79%	<0.001
8 Joint disease	19.41%	21.71%	<0.001
9. Neurological disease	9.81%	12.20%	<0.001
10. COVID-19	-	32.35%	<0.001
None of the above	33.89%	20.64%	<0.001

*—No COVID-19 disease occurred.

**Table 3:** Percentage of respondents in groups differing in their diet before the pandemic (2019–2020) and during the pandemic (2021–2022) (*p* < 0.05 is considered statistically significant).

What Meals Do You Eat Most Often?	Before the Pandemic 2019–2020 *n* = 17,000	During the Pandemic 2021–2022 *n* = 17,000	*p*-Value
Meals balanced according to the food pyramid	12.16%	10.64%	<0.001
Vegetarian meals	2.44%	2.27%	0.144
Vegan meals	0.19%	0.20%	0.662
Meat meals	13.51%	11.14%	<0.001
Gluten-free meals	0.42%	0.46%	0.485
Dairy-free meals	0.35%	0.35%	0.948
Carbohydrate-restricted meals	2.29%	2.56%	0.023
Reduced-sodium meals	3.75%	3.21%	<0.001
Other types of meals	0.89%	1.11%	0.005
I do not know	14.00%	18.07%	<0.001

**Table 4:** Percentage of respondents in groups differing in terms of diet and self-reported disease in 2021–2022.

DIET	Self-Reported Disease									
	Hypertension	Diabetes	Heart Disease	COPD	Allergy and Asthma	Depression	Cancer	Joint Disease	Neurological Disease	COVID-19
Meals balanced according to the food pyramid	6.45%	1.73%	2.55%	0.46%	4.40%	2.55%	1.40%	4.09%	2.10%	6.43%
Vegetarian meals	0.92%	0.20%	0.44%	0.05%	1.26%	1.00%	0.24%	0.74%	0.41%	1.56%
Vegan meals	0.09%	0.03%	0.05%	0.01%	0.14%	0.11%	0.03%	0.09%	0.05%	0.17%
Meat meals	9.57%	2.78%	3.53%	0.92%	4.31%	2.91%	1.44%	5.47%	2.85%	6.79%
Gluten-free meals	0.28%	0.12%	0.17%	0.03%	0.36%	0.21%	0.08%	0.26%	0.16%	0.32%
Dairy-free meals	0.25%	0.07%	0.12%	0.03%	0.25%	0.16%	0.06%	0.21%	0.13%	0.24%
Carbohydrate-restricted meals	2.13%	1.12%	0.81%	0.15%	1.09%	0.82%	0.38%	1.29%	0.64%	1.59%
Saodium-reduced meals	3.84%	1.03%	1.65%	0.31%	1.46%	0.99%	0.70%	2.21%	1.12%	1.96%
Another type of meals with limited carbohudrates	0.91%	0.32%	0.42%	0.09%	0.62%	0.48%	0.21%	0.70%	0.40%	0.74%
Another type of sodium-restricted meals	13.75%	3.98%	5.49%	1.35%	7.62%	5.91%	2.46%	8.99%	5.19%	11.41%

**Table 5:** Values of the regression coefficients for the correlation of diet, sociodemographic factors, and the presence of the COVID-19 pandemic with hypertension with predictors significant in the univariate analysis.

Hypertension	b	*p*	Beta	*p*	OR (95% CI)
	-	-	−7.573	<0.001	-
During the pandemic	0.12	<0.001	0.133	<0.001	1.14 (1.08–1.20)
Male	0.51	<0.001	0.303	<0.001	1.35 (1.28–1.43)
Age	0.07	<0.001	0.067	<0.001	1.07 (1.07–1.07)
Education level	−0.28	<0.001	−0.076	<0.001	0.93 (0.89–0.96)
Population of the place of residence	−0.03	<0.001	−0.015	0.014	0.99 (0.97–1.00)
BMI	0.14	<0.001	0.123	<0.001	1.13 (1.12–1.14)
Meals balanced according to the food pyramid	−0.36	<0.001	−0.143	<0.001	0.87 (0.81–0.93)
Vegetarian meals	−0.95	<0.001	−0.366	<0.001	0.69 (0.60–0.81)
Meat meals	0.27	<0.001	0.088	0.009	1.09 (1.02–1.17)
Reduced-sodium meals	0.69	<0.001	0.326	<0.001	1.38 (1.25–1.53)

b—univariate logistic regression coefficient, beta—multivariate logistic regression coefficient, *p*—significance level of regression coefficient, OR—odds ratio.

**Table 6:** Values of the regression coefficients for the correlation of diet and sociodemographic factors with COVID-19 disease with predictors significant in the univariate analysis.

COVID-19	Univariate		Multivariate		
	b	*p*	Beta	*p*	OR (95% CI)
	-	-	−1.461	<0.001	-
Male gender	−0.17	<0.001	−0.122	<0.001	0.88 (0.83–0.94)
Age	−0.01	<0.001	−0.011	<0.001	0.99 (0.99–0.99)
Married	0.14	<0.001	0.193	<0.001	1.24 (1.14–1.29)
Meals balanced according to the food pyramid	−0.05	0.131	−0.199	<0.001	0.82 (0.76–0.89)
Reduced-sodium meals	−0.29	<0.001	−0.333	<0.001	0.72 (0.63–0.82)

b—univariate logistic regression coefficient, beta—multivariate logistic regression coefficient, *p*—significance level of regression coefficient, OR—odds ratio.

**Table 7:** Values of the regression coefficients for the correlation of diet, sociodemographic factors, and the presence of the COVID-19 pandemic with allergies and asthma with predictors significant in the univariate analysis.

Allergies or Asthma	Univariate		Multivariate		
	b	*p*	Beta	*p*	OR (95% CI)
	-	-	−1.440	<0.001	-
During the pandemic	0.25	<0.001	0.258	<0.001	1.29 (1.23–1.37)
Male gender	−0.40	<0.001	−0.362	<0.001	0.70 (0.66–0.74)
Age	−0.01	<0.001	−0.013	<0.001	0.99 (0.98–0.99)
Married	−0.25	<0.001	−0.153	<0.001	0.86 (0.81–0.91)
Population of the place of residence	0.05	<0.001	0.036	<0.001	1.04 (1.02–1.05)
BMI	0.00	0.099	0.014	<0.001	1.01 (1.01–1.02)
Vegetarian meals	0.36	<0.001	0.198	0.001	1.22 (1.08–1.37)
Dairy-free meals	0.48	<0.001	0.412	0.005	1.51 (1.13–2.01)

b—univariate logistic regression coefficient, beta—multivariate logistic regression coefficient, *p*—significance level of regression coefficient, OR—odds ratio.

**Table 8:** Values of the regression coefficients for the correlation of diet, sociodemographic factors, and the presence of the COVID-19 pandemic with joint disease with predictors significant in the univariate analysis.

Joint Disease	Univariate		Multivariate		
	b	*p*	Beta	*p*	OR (95% CI)
	-	-	−4.351	<0.001	-
During the pandemic	0.14	<0.001	0.117	<0.001	1.12 (1.06–1.19)
Male gender	−0.33	<0.001	−0.548	<0.001	0.58 (0.54–0.61)
Age	0.05	<0.001	0.053	<0.001	1.05 (1.05–1.06)
Education level	−0.43	<0.001	−0.342	<0.001	0.71 (0.68–0.74)
Married	0.07	0.006	−0.060	0.045	0.94 (0.89–1.00)
Population of the place of residence	−0.04	<0.001	−0.017	0.010	0.98 (0.97–1.00)
BMI	0.06	<0.001	0.045	<0.001	1.05 (1.04–1.05)
Meals balanced according to the food pyramid	−0.27	<0.001	−0.102	0.006	0.90 (0.84–0.97)
Dairy-free meals	0.37	0.011	0.340	0.031	1.41 (1.03–1.91)

b—univariate logistic regression coefficient, beta—multivariate logistic regression coefficient, *p*—significance level of regression coefficient, OR—odds ratio.

**Table 9:** Values of the regression coefficients for the correlation of diet, sociodemographic factors, and the presence of the COVID-19 pandemic with depression with predictors significant in the univariate analysis.

Depression	Univariate		Multivariate		
	b	*p*	Beta	*p*	OR (95% CI)
	-	-	−1.326	<0.001	-
During the pandemic	0.42	<0.001	0.424	<0.001	1.53 (1.43–1.63)
Male gender	−0.59	<0.001	−0.556	<0.001	0.57 (0.53–0.62)
Age	−0.01	<0.001	−0.005	<0.001	0.99 (0.99–1.00)
Education level	−0.05	<0.001	−0.141	<0.001	0.87 (0.83–0.91)
Married	−0.50	<0.001	−0.396	<0.001	0.67 (0.63–0.72)
Population of the place of residence	0.05	<0.001	0.053	<0.001	1.05 (1.04–1.07)
Meals balanced according to the food pyramid	−0.24	<0.001	−0.185	<0.001	0.83 (0.76–0.91)
Vegetarian meals	0.46	<0.001	0.250	<0.001	1.28 (1.12–1.47)

b—univariate logistic regression coefficient, beta—multivariate logistic regression coefficient, *p*—significance level of regression coefficient, OR—odds ratio.

**Table 10:** Values of the regression coefficients for the correlation of diet, sociodemographic factors, and the presence of the COVID-19 pandemic with heart disease with predictors significant in the univariate analysis.

Heart Disease	Univariate		Multivariate		
	b	*p*	Beta	*p*	OR (95% CI)
	-	-	−5.424	<0.001	-
During the pandemic	0.18	<0.001	0.164	<0.001	1.18 (1.10–1.26)
Age	0.06	<0.001	0.058	<0.001	1.06 (1.06–1.06)
Education level	−0.28	<0.001	−0.180	<0.001	0.84 (0.80–0.88)
BMI	0.05	<0.001	0.023	<0.001	1.02 (1.02–1.03)
Meals balanced according to the food pyramid	−0.26	<0.001	−0.116	0.016	0.89 (0.81–0.98)
Gluten-free meals	0.42	0.006	0.505	0.002	1.66 (1.21–2.28)
Dairy-free meals	0.40	0.019	0.529	0.004	1.70 (1.19–2.43)
Reduced-sodium meals	0.53	<0.001	0.182	0.003	1.20 (1.06–1.35)

b—univariate logistic regression coefficient, beta—multivariate logistic regression coefficient, *p*—significance level of regression coefficient, OR—odds ratio.

**Table 11:** Values of the regression coefficients for the correlation of diet, sociodemographic factors, and the presence of the COVID-19 pandemic with neurological disease with predictors significant in the univariate analysis.

Neurological Disease	Univariate		Multivariate		
	b	*p*	Beta	*p*	OR (95% CI)
	-	-	−2.548	<0.001	-
During pandemic	0.25	<0.001	0.220	<0.001	1.25 (1.16–1.34)
Male gender	−0.30	<0.001	−0.377	<0.001	0.69 (0.64–0.74)
Age	0.03	<0.001	0.026	<0.001	1.03 (1.02–1.03)
Education level	−0.42	<0.001	−0.365	<0.001	0.69 (0.66–0.73)
Married	−0.06	0.092	−0.126	0.001	0.88 (0.82–0.95)
Population of the place of residence	−0.04	<0.001	−0.018	0.027	0.98 (0.97–1.00)
Meals balanced according to the food pyramid	−0.32	<0.001	−0.207	<0.001	0.81 (0.74–0.89)
Vegetarian meals	−0.50	<0.001	−0.329	0.001	0.72 (0.59–0.88)
Dairy-free meals	0.55	0.001	0.456	0.009	1.58 (1.12–2.22)

b—univariate logistic regression coefficient, beta—multivariate logistic regression coefficient, *p*—significance level of regression coefficient, OR—odds ratio.

**Table 12:** Values of the regression coefficients for the correlation of diet, sociodemographic factors, and the presence of the COVID-19 pandemic with diabetes with predictors significant in the univariate analysis.

Diabetes	Univariate		Multivariate		
	b	*p*	Beta	*p*	OR (95% CI)
	-	-	−8.339	<0.001	-
During the pandemic	0.17	<0.001	0.146	<0.001	1.16 (1.07–1.25)
Male gender	0.38	<0.001	0.184	<0.001	1.20 (1.11–1.30)
Age	0.06	<0.001	0.054	<0.001	1.06 (1.05–1.06)
Education level	−0.31	<0.001	−0.190	<0.001	0.83 (0.78–0.87)
Married	0.25	<0.001	0.093	0.028	1.10 (1.01–1.19)
Population of the place of residence	0.00	0.826	0.020	0.031	1.02 (1.00–1.04)
BMI	0.12	<0.001	0.114	<0.001	1.12 (1.11–1.13)
Vegetarian meals	−0.99	<0.001	−0.315	0.026	0.73 (0.55–0.96)
Dairy-free meals	−1.22	0.001	−1.078	0.007	0.34 (0.16–0.74)

b—univariate logistic regression coefficient, beta—multivariate logistic regression coefficient, *p*—significance level of regression coefficient, OR—odds ratio.

**Table 13:** Values of the regression coefficients for the correlation of diet, sociodemographic factors, and the presence of the COVID-19 pandemic with cancer with predictors significant in the univariate analysis.

Cancer	Univariate		Multivariate		
	b	*p*	Beta	*p*	OR (95% CI)
	-	-	−6.025	<0.001	-
During the pandemic	0.21	<0.001	0.199	<0.001	1.22 (1.11–1.34)
Male gender	−0.31	<0.001	−0.456	<0.001	0.63 (0.57–0.70)
Age	0.05	<0.001	0.053	<0.001	1.05 (1.05–1.06)
Population of the place of residence	0.02	0.042	0.035	0.002	1.04 (1.01–1.06)

b—univariate logistic regression coefficient, beta—multivariate logistic regression coefficient, *p*—significance level of regression coefficient, OR—odds ratio.

**Table 14:** Values of the regression coefficients for the correlation of diet, sociodemographic factors, and the presence of the COVID-19 pandemic with COPD with predictors significant in the univariate analysis.

Chronic Obstructive Pulmonary Disease (COPD)	Univariate		Multivariate		
	b	*p*	Beta	*p*	OR (95% CI)
	-	-	−6.856	<0.001	-
Age	0.07	<0.001	0.071	<0.001	1.07 (1.07–1.08)
Education level	−0.57	<0.001	−0.495	<0.001	0.61 (0.55–0.67)
BMI	0.05	<0.001	0.015	0.037	1.02 (1.00–1.03)
Meals balanced according to the food pyramid	−0.78	<0.001	−0.658	<0.001	0.52 (0.41–0.65)

b—univariate logistic regression coefficient, beta—multivariate logistic regression coefficient, *p*—significance level of regression coefficient, OR—odds ratio.

## Data Availability

Data supporting the reported results can be found at https://narodowytestzdrowia.medonet.pl/ (accessed on 18 July 2023).

## Supplementary Materials

The following supporting information can be downloaded at: https://www.mdpi.com/article/10.3390/nu15183947/s1, Table S1: The questionnaire used in the study regarding diet in the Polish population—translated from Polish; Table S2: Sociodemographic factors of the respondents (*p* < 0.05); Table S3: Percentage of observed diseases among our study population divided into groups depending on when the data were self-reported: before the COVID-19 pandemic—2019–2020 and during the COVID-19 pandemic (2021–2022; *p* < 0.05 acts as significant difference); Table S4: Number (percentage) of respondents in groups differing in the frequency of different types of food consumed (in 2021–2022).
