# Supplementary material for: The Impact of Different Types of Diet on the Prevention of Diseases among Polish Inhabitants, Including COVID-19 Disease
Source: Nutrients. 2023 Sep 12;15(18):3947. doi: 10.3390/nu15183947 (PMC10535510; doi:10.3390/nu15183947)
Supplement: Supplementary file 1 [file nutrients-15-03947-s001.zip › nutrients-2562515-supplementary.pdf]

Table S1: Questionnaire used in this study:

Q1) What meals do you eat most often?

A. Meals balanced according to the food pyramid
B. Vegetarian meals
C. Vegan meals
D. Meat meals
E. Gluten-free meals
F. Dairy-free meals
G. Carbohydrate-restricted meals
H. Sodium-reduced meals
I. Another type of meals with limited carbohydrates
J. Another type of sodium-restricted meals

Q2) How often do you eat red meat?

A. Never
B. Once or twice a month
C. One to three times a week
D. Everyday

Q3) For what reason do you often eat meat?

- **A.** I have no ideas for preparing meatless meals
- **B.** Meat meals is better for my health
- **C.** I like eating meat
- **D.** I can have enough to eat more while consuming meat than other products
- **E.** Most meals should contain meat
- **F.** None of the above

Q4) Have you ever been on a slimming diet?
A. Yes, I am on a slimming diet all the time
B. Yes, at least once in the last year I was on a slimming diet
C. Yes, I was on a slimming diet
D. No, I've never been on a slimming diet

Q5) How often do you eat junk-food products (hamburgers, fries, hot dogs, etc.)?

A. Everyday
B. Several times a week
C. Once a week
D. Several times a month
E. Once a month
F. Less often than once a month
G. Never

Q6) How often do you drink sweetened, carbonated or non-carbonated beverages?

A. Everyday

B. Several times a week
C. Once a week
D. Several times a month
E. Once a month
F. Less often than once a month
G. Never

Q7) How often do you drink energy drinks?

A. Three or more times per day
B. One or two times per day
C. Several times a week
D. Once a week
E. Several times a month
F. Once a month
G. Less often than once a month
H. Never

Q8) How often do you eat vegetables?

A. Everyday
B. Several times a week
C. Once a week
D. Several times a month
E. Once a month
F. Less often than once a month
G. Never

Q9) How often do you eat fruit?

A. Everyday
B. Several times a week
C. Once a week
D. Several times a month
E. Once a month
F. Less often than once a month
G. Never

Q10A) Have you been taking dietary supplements or other over-the-counter drugs last year?

A. Yes, everyday
B. Yes, several times a week
C. Yes, once a week
D. Yes, several times a month
E. Yes, once a month
F. Yes, less often than once a month
G. Yes, only for a limited time
H. No

Q10B) What kind and for why did you take dietary supplements or other over-the-counter drugs last year?

A. Multivitamin preparations
B. To improve the condition of hair and/or skin
C. To improve memory and concentration
D. To improve eyesight and visual comfort
E. To reduce body weight
F. To improve digestion or reduce ailments
G. To improve your mood and well-being
H. To alleviate problems with falling asleep
I. To improve potency
J. Others

Table S2: Sociodemographic factors of the respondents (p<0.05).

Features (variables)	2019-2020<br>N = 376102	2021-2022<br>N = 200000
Gender:		
Female	58,3%	59,6%
Male	41,7%	40,4%
Age (years old)		
M ± SD	48 ± 15	53 ± 15
Me [Q1; Q3[	47 [37; 60]	54 [42; 65]
Min – Max	18 - 99	18 – 99
Education:		
Primary	1,2%	2,0%
lower secondary	1,1%	0,8%
basic vocational	8,2%	10,8%
Secondary	28,8%	29,7%
post-secondary	10,4%	10,6%
bachelor's degree	11,0%	9,5%
master’s degree	39,3%	36,6%
Place of residence:		
village	21,3%	22,1%
town, less than 19,000 inhabitants	11,3%	11,9%
town, between 20,000 to 49,000 inhabitants	14,2%	15,4%
town, between 50,000 to 99,000 inhabitants	11,1%	11,6%
town, between 100,000 to 199,000 inhabitants	10,4%	11,0%
town, between 200,000 to 499,000 inhabitants	10,2%	10,6%
town, more than 500,000 inhabitants	21,6%	17,3%
Voivodeship:		
Lower Silesia	8,0%	8,4%
Kuyavian-Pomeranian	4,5%	5,2%
Lublin	4,2%	4,2%
Lubuskie	2,5%	2,7%
Lodz	6,5%	6,1%
Lesser Poland	10,4%	8,7%
Masovian	16,5%	15,8%
Opole	2,3%	2,6%
Subcarpathian	5,0%	4,8%
Podlaskie	2,4%	2,4%
Pomeranian	5,3%	5,8%
Silesian	14,2%	14,1%
Świętokrzyskie	2,7%	2,8%
Warmian-Masurian Voivodeship	2,8%	3,0%
Greater Poland	8,6%	8,8%
West Pomeranian	4,1%	4,6%
Body height (cm)		
M ± SD	170 ± 9	170 ± 9
Me [Q1; Q3]	170 [164; 177]	169 [164; 176]
Min – Max	120 - 215	120 - 215

Body mass (kg)		
M ± SD	79 ± 18	79 ± 18
Me [Q1; Q3[	77 [65; 90]	77 [66; 90]
Min - Max	30 - 230	31 - 210
BMI (kg/m^2):		
M ± SD	26,9 ± 5,1	27,2 ± 5,1
Me [Q1; Q3[	26 [23; 30]	27 [24; 30]
Min - Max	12 - 60	12 - 60

Table S3. Percentage of observed diseases among our study population divided into groups depending on when the data were self-reported: before the COVID-19 pandemic – 2019-2020, and during the COVID-19 pandemic (2021-2022; $p<0.05$ acts as significant different)

Observed diseases	2019-2020 N=376,102	2021-2022 N=200,000	p-value
1. Hypertension	29,6%	38,2%	<0,001
2. Diabetes	7,7%	11,4%	<0,001
3. Heart disease	10,2%	15,2%	<0,001
4. Chronic obstructive pulmonary disease (COPD)	2,1%	3,4%	<0,001
5. Allergy or asthma	18,2%	21,5%	<0,001
6. Depression	10,1%	15,1%	<0,001
7. Cancer disease	4,3%	7,0%	<0,001
8. Joint disease	17,9%	24,0%	<0,001
9. Neurological disease	9,2%	13,0%	<0,001
10. COVID-19	-	31,2%	-
None of the above	38,4%*	19,2%	<0,001

* - no COVID-19 disease occurred

Table S4: Number (percentage) of respondents in groups differing in the frequency of different types of consumed food (in 2021-2022).

**What meals do you eat most often?**	*n*	(%)
Meals balanced according to the food pyramid	40922	20.5
Vegetarian meals	9154	4.6
Vegan meals	956	0.5
Meat meals	44254	22.1
Gluten-free meals	1899	0.9
Dairy-free meals	1451	0.7
Carbohydrate-restricted meals	9992	5.0
Sodium-reduced meals	14266	7.1
Another type of meals with limited carbohydrates	4770	2.4
Another type of sodium-restricted meals	72336	36.2
**How often do you eat red meat?**	*n*	(%)
Never	24369	12,2%
Once or twice a month	82333	41,2%
One to three times a week	73613	36,8%
Everyday	9561	4,8%
**For what reasons do you often eat meat?**	*n*	(%)
I have no ideas for preparing meatless meals	7433	3,7%
Meat meals is better for mu health	1869	0,9%
I like eating meat	26404	13,2%
I can have enough to eat more while consuming meat than other products	9146	4,6%
Most meals should contain meat	6972	3,5%
Non of the above		78,1%
**Have you ever been on a slimming diet?**		
Yes, I am on a slimming diet all the time	9156	4,6%
Yes, at least once in the last year I was on a slimming diet	21362	10,7%
Yes, I was on a slimming diet	56919	28,5%
No, I've never been on a slimming diet	112563	56,3%
**How often do you eat junk-food products (hamburgers, fries, hot dogs, etc.)?**		
Everyday	245	0,12%
Several times a week	2884	1,44%
Once a week	7232	3,62%
Several times a month	26513	13,26%
Once a month	28685	14,34%
Less often than once a month	86395	43,20%
Never	48046	24,02%
**How often do you drink sweetened, carbonated or non-carbonated beverages?**		
Everyday	20014	10,0%
Several times a week	19143	9,6%
Once a week	10548	5,3%
Several times a month	27567	13,8%
Once a month	14151	7,1%
Less often than once a month	53135	26,6%
Never	55442	27,7%
**How often do you drink energy drinks?**		

Three or more times per day	443	0,2%
One or two times per day	1246	0,6%
Several times per week	3490	1,7%
Once a week	2289	1,1%
Several Times per month	6093	3,0%
Once a month	4836	2,4%
Less often than once a month	24649	12,3%
Never	156954	78,5%
**How often do you eat vegetables?**		
Everyday	70600	35,3%
Several times a week	84200	42,1%
Once a week	16400	8,2%
Several times a month	20400	10,2%
Once a month	3200	1,6%
Less often than once a month	4200	2,1%
Never	800	0,4%
**How often do you eat fruits?**		
Everyday	84400	42,2%
Several times a week	72400	36,2%
Once a week	15200	7,6%
Several times a month	18400	9,2%
Once a month	3600	1,8%
Less often than once a month	4800	2,4%
Never	1000	0,5%
**Have you been taking dietary supplements or other over-the-counter drugs last year?**		
Yes, everyday	50000	25,0%
Yes, several times a week	26600	13,3%
Yes, once a week	4000	2,0%
Yes, several times a month	10000	5,0%
Yes, once a month	2600	1,3%
Yes, less often than once a month	6000	3,0%
Yes, only for a limited time	20600	10,3%
No	80200	40,1%
**What kind and for why did you take dietary supplements or other over-the-counter drugs last year?**		
Multivitamin preparations	70400	35,2%
To improve the condition of hair and/or skin	28000	14,0%
To improve memory and concentration	13200	6,6%
To improve eyesight and visual comfort	9400	4,7%
To reduce body weight	10000	5,0%
To improve digestion or reduce ailments	24800	12,4%
To improve your mood and well-being	12400	6,2%
To alleviate problems with falling asleep	15400	7,7%
To improve potency	2800	1,4%
Others	16600	8,3%
